# What Drives Water Utility Selection of Pricing Methods? Evidence from California

**DOI:** 10.1007/s11269-021-03018-8

**Published:** 2021-11-04

**Authors:** M. Allaire, A. Dinar

**Affiliations:** 1 Department of Urban Planning and Public Policy, University of California, Irvine, USA; 2 School of Public Policy, University of California, Riverside, USA

**Keywords:** Water rates, Water pricing, Conservation, Water utility, Adoption, California

## Abstract

Water pricing is a demand management strategy to address the looming challenge of greater water scarcity in arid regions. Much of the literature on residential water rates focuses on evaluating the impact of pricing on household conservation. A separate, but rarely addressed question is what motivates a water utility to select a particular rate structure and the timing of doing so. We assess utilities’ decisions to adopt pro-conservation rate structures, such as increasing block rates and water budget rates. We develop a conceptual model of utility decision-making regarding the transition to pro-conservation rates and apply it to California.

We examine the relationship between rate adoption and characteristics of utilities and customers using logistic regression and a balanced panel dataset of 323 California water systems from 2006-2015. We find a notable shift towards pro-conservation rates, which 71% of California utilities had by 2015, compared to 44% in 2006. Capacity factors associated with adoption include size of service population and customer income level, while motivating factors include peer adoption, greater customer engagement, and special district governance. Overall, this study provides insight into barriers to pro-conservation pricing, which can inform policies to enable transitions and advance conservation goals.

**Supplementary Information:**

The online version contains supplementary material available at 10.1007/s11269-021-03018-8.

## Introduction

The looming challenge of water scarcity in many arid regions worldwide (Cosgrove and Loucks 2015) has led water suppliers to implement various supply augmentation and demand-side management strategies. Retail water pricing and conservation have especially been emphasized. To date, the bulk of the economic literature on retail water rates has focused on evaluation of the impact of pricing on household water use (e.g., Arbués et al. 2003; Brent and Ward 2019; Dalhuisen et al. 2003; Wichman 2014).

A separate, yet rarely addressed question of importance to policy makers, is what motivates a water utility in selecting a given rate structure and what determines the timing of that decision. Few studies address this research area and analyze key drivers that influence water utility selection of rate structures (e.g. Boyer et al. 2012; Boyer et al. 2014; Teodoro 2010; Gurung and Martínez-Espiñeira 2019).

In this paper, we examine what motivates utilities to adopt rate structures that can motivate conservation and what can help or hinder this transition. Why do some utilities adopt new rate structures while others do not? Our study sheds light on utility decision processes and what drives early vs. late adoption.

A notable contribution is our development of an analytical framework that describes adoption of pro-conservation water rates (PCWRs). PCWRs are volumetric rates that send a price signal to incentivize reduced use by charging higher prices for greater levels of consumption; examples include increasing block rates and water budget rates. We also frame PCWR adoption as a diffusion process and empirically estimate PCWR diffusion over time. Understanding how quickly PCWRs spread across utilities can better inform conservation targets and regulatory assessment of conservation policy performance. Our methods offer several advances by utilizing panel data to examine rate transitions, rather than being limited to cross-sectional comparison, as in previous studies. In addition, we focus on uptake of PCWRs in California over a decade, 2006 to 2015. This is a longer time horizon than most past literature and captures a more complete period of the rate diffusion process. Lastly, we consider factors not considered in previous empirical analyses, such as peer effects, customer complaints, and water rights seniority. The relevancy of results extends beyond California; utilities across the U.S. face increasing pressures of scarcity, whether prolonged or seasonal.

The remainder of the article is organized as follows: Next we provide background on PCWRs. In the third section, we review relevant literature on utility behavior and rate adoption. In the fourth section, we develop the analytical framework used to derive our empirical hypotheses. Data and methods are described in the fifth and sixth sections, followed by a discussion of results in the seventh section.

## Pro-conservation Water Rates

Common types of pro-conservation pricing structures are increasing block rates (IBR) and water budget rates. These incentivize reduced use since the marginal price faced by consumers increases with demand (Pinto and Marques 2015; Kenney et al. 2008; Olmstead et al. 2007). We categorize rate structures based on their volumetric component; any pricing structure can also have a fixed charge component.

Several studies find that IBRs cause consumers to become more sensitive to price changes, which suggests that this rate structure is an effective tool for conservation (Dalhuisen et al. 2003; Olmstead et al. 2007). Yet, this pricing structure might have little effect on conversation if customers respond to average, rather than marginal price (Ito 2014). In addition, only the highest volume users might face a strong conservation price signal (Brandes et al. 2010; Renzetti et al. 2015). A barrier for utility adoption of IBRs can be concerns over greater variability in revenue compared to uniform rates (Dinar and Ash 2015). Revenue variability is influenced by rate design, including number of blocks and price differentials between blocks.

A variant of IBRs is a water budget rate. Within this block rate structure, volumetric tiers are tailored to the characteristics of an individual customer. Each customer is assigned a water use budget, typically based on number of household members, landscaped area, and environmental conditions. If customers exceed this usage level, then a considerably higher volumetric price is charged for ‘excess’ use. This structure offers the potential advantage of encouraging conservation while obtaining more stable revenues, since customers do not face a higher marginal price if they remain within their allotted usage level. Implementation of this structure can be data intensive and often requires extensive public outreach.

## Utility Adoption of PCWR: Literature Review

Few studies address why utilities transition to new rate structures. Much of the water conservation literature focusses on how residential users respond to changes in water price. No formal model describes water utility behavior and decision-making related to PCWR adoption. Of the few empirical studies to address water rate adoption, most are limited to cross-sectional analysis (Gurung and Martínez-Espiñeira 2019; Hanak 2005; Hewitt 2000; Montginoul 2007; Mullin 2008; Reynaud et al. 2005; Teodoro 2010). Cross-sectional studies can address factors associated with rate structures observed in a given year, but not those associated with transitions from one rate to another. Several cross-sectional studies have examined water system characteristics associated with U.S. utilities choosing an IBR, both nationwide (Hewitt 2000; Mullin 2008; Teodoro 2010) and in California (Hanak 2005). Beyond the U.S., cross-sectional analyses have also been conducted in Canada (Gurung and Martínez-Espiñeira 2019; Reynaud et al. 2005) and France (Montginoul 2007). Both Gurung and Martínez-Espiñeira (2019) and Reynaud et al. (2005) develop multinomial models to examine determinants of rate structure choice in the years 2009 and 2001, respectively. Gurung and Martínez-Espiñeira (2019) analyze physical characteristics of the utility and find that considerable unexplained variability that might be attributable to conservation goals, fairness, and political acceptability. In contrast to the U.S. and Canada, Montginoul (2007) finds that IBRs are rarely adopted in France.

Panel data analyses have emerged more recently. Only two prior studies (Boyer et al. 2012; Boyer et al. 2014) have conducted regression analysis using a panel dataset of rate adoption. These two studies addressed rate changes over a five-year period, 2005-2009, for 695 utilities in the southern United States. Based on a survey of utility managers, stated reasons for rate changes included conservation, equitable customer prices, and revenue concerns (Boyer et al. 2012). Price-based conservation was more likely at utilities with larger population growth, reliance on purchased water, and planned infrastructure upgrades to meet future demand (Boyer et al. 2014). An additional study that utilizes panel data is Gaur and Diagne (2017), yet the analysis is limited to summary statistics. This study provides a county-level summary of water rate structures in California from 2003-2015 (Gaur and Diagne 2017). In contrast to Gaur and Diagne (2017), we go beyond describing shifts in rates; our study analyzes underlying drivers of rate changes.

We contribute to the small, yet growing, body of empirical literature on the selection process for rate structures. Our study uses panel data analysis to examine rate transitions, rather than being limited to cross-sectional comparison. We consider drivers not considered in previous empirical analyses, such as peer effects, customer complaints, and seniority of water rights. Compared to other studies, we assess rate adoption decisions over a long time horizon – 10 years. This allows our analysis to capture a more complete portion of the rate diffusion process. Past research has relied on short study periods and surveys of relatively small samples of utilities. By utilizing secondary data from multiple sources, we develop a higher-quality dataset and a more representative sample of utilities.

## Analytical Framework of Utility Adoption of Pro-conservation Rates

Our analytical framework includes two aspects of adoption. First, we describe the diffusion process for PCWRs. Then, we describe the drivers of PCWR adoption by water utilities, using results from previous studies that explain water utility behavior. We discuss historical uptake of PCWRs in California and drivers for their adoption (Supplementary Information (SI), Text S1). Lastly, we introduce formal models of the relationship between explanatory variables and the adoption decision.

### The Diffusion Process

**Fig. 1:**
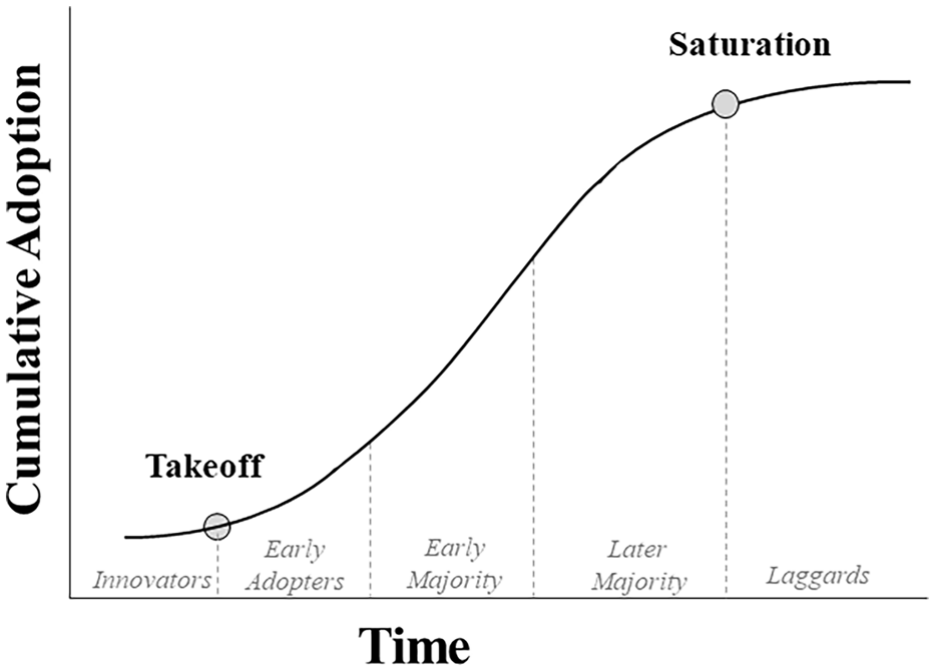
The Rogers’ innovation diffusion curve. Adapted from Rogers (2003)

We frame rate adoption as the diffusion of a policy innovation over time, following Rogers (2003) framework on innovation diffusion. We refer to PCWRs as an innovation and to water utilities as adopters. Our analysis focuses on organizational behavior, rather than individual behavior. Several studies suggest that organization adoption aligns with individual behavior described in Rogers’ framework (Berry and Berry 2018; Dearing and Cox 2018). In the technology adoption literature, innovation diffusion is traditionally represented by an S-curve (Rogers 2003). Distinct stages of the diffusion process are depicted on this S-curve, which begins with ‘take-off’ and ends with ‘saturation’. Individual adopters can be characterized as having different tendencies and abilities to adopt new innovations, based on how early they transition. Categories of adoption range from early adopters to laggards (Fig. 1), and there are also those who never adopt.

We present an adopter function, which represents cumulative diffusion of an innovation over time. This has the general form:

1 $$\frac{\partial {{n}_{t}}/{N}}{\partial t}=\varnothing \frac{{n}_{t}}{N}(\overline{N }-\frac{{n}_{t}}{N})$$

where *n*_*t*_ is the cumulative number of utilities that adopted a PCWR by year *t*; *N* is the total number of utilities; $\overline{N }$ is the potential percentage of utilities that will adopt; and $\varnothing$ is a parameter representing the rate of adoption. As the portion of adopters ($\frac{{n}_{t}}{N}$) in any year *t* increases, the portion of potential adopters ($\overline{N }-\frac{{n}_{t}}{N}$) decreases. Therefore, the diffusion of PCWR is expected to slow as $\frac{{n}_{t}}{N}$ grows and reach a steady state; this forms a sigmoid cumulative density function. Solving for $\frac{{n}_{t}}{N}$ in Eq. (1) results in:

2 $$\frac{{n}_{t}}{N}=\frac{1}{1+{e}^{-{\beta }_{0}-\varnothing \overline{N}t} }$$

where ${\beta }_{0}$ is the constant of integration. We estimate the adopter function for PCWRs in California (Sect. 7.2); anticipating the timing of PCWR uptake can aid in estimating expected water use and inform water management decisions.

### Adoption Drivers

We categorize drivers of rate adoption as external or internal to the utility, based on a review of the literature. External factors include ‘shocks’ such as changes in policy, climate conditions (Pinto and Marques 2015), and drought events (Kwon and Bailey 2019). Internal factors reflect characteristics of an individual utility. A diagram of adoption drivers is provided in Fig. S1.

#### External Factors

External factors affect multiple utilities and include drought events, long-term climatic conditions, and substantial policy changes. Severe drought conditions have been found to be associated with adoption of PCWRs (Kwon and Bailey 2019). In addition, PCWRs are more likely to be adopted in locations with less precipitation (Pinto and Marques 2015; Teodoro 2010) and higher temperature (Mullin 2008; Pinto and Marques 2015). In our analytical model (Sect. 4.3), we expect that drought and arid conditions will increase the likelihood of PCWR adoption (Kwon and Bailey 2019), since increasing block rates are generally considered to encourage conservation (Kenney et al. 2008; Olmstead et al. 2007). Laws, regulations, and policies can also influence rate adoption decisions and can occur at multiple levels – Federal, state, county, and water district. Such policies include those specifically targeted at conservation as well as those related to rate setting and metering. We anticipate that such policies will increase the likelihood of PCWR adoption. Certain rate structure components might be required as pre-requisite for government grants and loans (Boyer et al. 2012).

#### Internal Factors

Internal drivers can be further divided into two sub-categories: capacity and motivating factors. Capacity factors are necessary conditions, which must be in place for a transition to occur, but these alone will not result in adoption. Motivating factors are the sufficient conditions that will allow the transition to occur, given that the capacity factors are in place.

#### Capacity Factors

Capacity factors characterize the technical, managerial, and financial ability of the utility to introduce rate reforms. These factors include system size, financial wellbeing, extent of metering, and demand hardening. Larger utilities, as measured by service population or produced water volume, are more likely to adopt PCWRs (Hewitt 2000; Hanak 2005; Teodoro 2010; Boyer et al. 2012). Larger utilities have greater managerial capacity to develop rate cases, accurately predict revenue, and administer more complex rate structures. Larger utilities also tend to be in better financial health and can spread fixed costs across a more extensive customer base. Financial capacity is also influenced by customer income levels – those serving higher-income customers might be better able to secure an adequate revenue and external financing.

Water metering within a service area is a necessary condition for any volumetric rate. Several major U.S. cities lacked universal metering until the 1980s; prior to this time, fixed and uniform rates were prevalent. Since then, IBRs have spread rapidly (Olmstead et al. 2007).

Level of water use per capita can also be considered a capacity factor. If a utility already has achieved large reductions in water usage, then there might not be much room for further reductions. Demand hardening can cause price elasticity of water demand to decline to near zero (Kenney et al. 2008). Yet, if additional conservation is possible, then there is past dependency in utility decisions (Montginoul 2007); utilities that have implemented conservation policies might be more likely to adopt PCWRs .

#### Motivating Factors

Motivating factors are the stimulus for adopting new pricing methods. These include reliance on water imports, expected future infrastructure investment, revenue considerations, utility governance, and social interactions. Water purchased from other utilities has been found to be associated with adoption of PCWRs (Boyer et al. 2014). During drought periods, imports can become costly or be drastically reduced for junior water rights-holders. Thus, utilities have an incentive to conserve and diversify their raw water sources away from imports, particularly if they are junior rights-holders.

An additional motivating factor is expected need for infrastructure investment, due to anticipated changes. Utilities anticipating population growth have been found to be more likely to adopt PCWRs (Boyer et al. 2014) since conservation can reduce the cost of new infrastructure due to delayed or downsized capacity expansions. Investor-owned utilities might be less likely to have this motivation since in the U.S. they tend not to own the water infrastructure they operate – long-term contracts are more common than full divestiture. Operators on limited term contracts are less likely to make major capital investments.

A third motivating factor is revenue. Some utilities adopt PCWRs in order to increase revenue and cover growing regulatory costs (Boyer et al. 2012). Yet, a barrier to PCWRs is concerns about revenue variability. Investor-owned utilities might be less likely to adopt PCWRs due to these concerns. Water budget rates offer greater revenue stability over IBRs.

Utility governance also plays a role. Ownership is one aspect of governance; publicly and privately-owned utilities may face differing incentives. Furthermore, publicly-owned utilities can either be a general-purpose government (e.g. municipality, county) or a special district, which is an independent government established to provide specific services.^1^ Based on public choice theory, specialized governments can be more transparent and responsive to majority opinion (Mullin 2008; Ostrom et al. 1961). IBRs are likely attractive for the median household, since the right skew of residential water demand means that relatively few large water users will bear a larger portion of service costs (Mullin 2008). Under an IBR, a majority of households are expected to face lower water bills, compared to a uniform rate designed to collect the same revenue (Chestnutt et al. 1997). Elected officials in special districts are hypothesized to be more responsive to majority opinion regarding water rates since pricing is a salient issue and constituents can their preferences on water pricing from other local issues (Mullin 2008).

Social interactions include customer pressure and peer effects. Customers can exert pressure through water board meetings and local elections. Effective engagement with customers can increase understanding and acceptance of PCWRs. Peer effects can influence decisions if utilities compare and imitate water rate structures of neighboring utilities (e.g., (Pinto and Marques 2015). PCWR adoption by neighboring utilities can allow for learning and changes in norms regarding rate structure types. In addition, utilities are more likely to adopt a PCWR if staff participate in professional development activities (Teodoro 2010), which include attending professional conferences, reading technical reports, and consulting with peers on policy issues.

We expect that utilities more likely to adopt PCWRs are those with imported water supply, junior water rights, governance structures that allow customers to directly elect utility officials, and greater social interactions with customers.

### A Simplified Analytical Model

We identify two types of decisions taken by a water utility: (1) whether or not to adopt PCWR, and (2) the timing of PCWR adoption (*Tm*). The adoption decision (*Ad*) is a dichotomous decision. Subject to adoption of PCWR, the timing decision (*Tm*) can be measured in relative terms of early or late time periods. We only present the adoption decision model, given that both *Ad* and *Tm* are influenced by a similar set of explanatory variables. Our models make no assertions regarding welfare changes due to PCWR adoption. The model representing the adoption behavior of a water utility includes the effect of external (*Ex*) and internal (*In*) factors:

3 $$Ad =f(Ex, In)$$

The function for external factors is:

4 $$Ex=h(Cl, La)$$

where *Cl* represents arid climate factors such as drought events and long-term hot or dry climate conditions, while *La* represents changes to laws, regulations, and policies. Based on previous literature, it is expected that $\frac{\partial Ad}{\partial Cl}\ge 0$, while $\frac{\partial Ad}{\partial La}\frac{>}{<} 0$, depending on the specific policy.

The function for internal factors is:

5 $$In=j(Ca, Mo)$$

where *Ca* are capacity factors and *Mo* are motivating factors for the utility. The first component representing capacity can be written as:

6 $$Ca=k(Tc)$$

where *Tc* is total capacity of the water utility. *Tc* can be expressed as a combination of technical, managerial, and financial capacity. Utility capacity can be indicated by size of service population and customer income levels. Our expectation is: $\frac{\partial Ad}{\partial Tc}\ge 0$.

The second component of the function for internal factors function is given by:

7 $$Mo=m(Wi, Gv, Sc | Ca)$$

where *Wi* is reliance on water imports and/or junior water rights, *Gv* is utility governance, and *Sc* is social interactions such as interaction between the utility and customers as well as adoption of PCWRs by neighboring utilities. These expectations can be summarized as: $\frac{\partial Ad}{\partial Wi}\ge 0; \frac{\partial Ad}{\partial Gv}\ge 0; \frac{\partial Ad}{\partial Sc}\ge 0$.

Incorporating Eqs. (4)-(7) into equation 3 yields:

8 $$Ad=f[h(Cl, La),\mathrm{ j}(k(Tc)), m(Wi, Gv, Sc | k(Tc))]$$

To obtain the overall effect of each of the variables on the likelihood to adopt PCWRs, while holding all other variables constant, we differentiate Eq. (8) with respect to each variable. We obtain the following differential equation chain:

9 $$\frac{dAd}{dCl}=\frac{\partial Ad}{\partial f}\frac{\partial f}{\partial h}\frac{\partial h}{\partial Cl}\ge 0$$

10 $$\frac{dAd}{dLa}=\frac{\partial Ad}{\partial f}\frac{\partial f}{\partial h}\frac{\partial h}{\partial La}\frac{>}{<}0$$

11 $$\frac{dAd}{dTc}=\frac{\partial Ad}{\partial f}\frac{\partial f}{\partial j}\frac{\partial j}{\partial k}\frac{\partial k}{\partial Tc}+\frac{\partial j}{\partial m}\frac{\partial m}{\partial k}\frac{\partial k}{\partial Tc}\ge 0$$

12 $$\frac{dAd}{dWi}=\frac{\partial Ad}{\partial f}\frac{\partial f}{\partial m}\frac{\partial m}{\partial Wi}\ge 0$$

13 $$\frac{dAd}{dGv}=\frac{\partial Ad}{\partial f}\frac{\partial f}{\partial m}\frac{\partial m}{\partial Gv}\ge 0$$

14 $$\frac{dAd}{dSc}=\frac{\partial Ad}{\partial f}\frac{\partial f}{\partial m}\frac{\partial m}{\partial Sc}\ge 0$$

Eqs. (9), (10), (11), (12), (13) and (14) provide the set of hypotheses for the empirical estimate, presented in Sect. 6.

## Data

We create a balanced panel of 323 community water systems (CWS) for the years 2006-2015 to assess drivers of utility decisions to adopt PCWRs. This panel dataset is more representative of CWS in California, compared to past water rate surveys. It was compiled using secondary datasets and a survey targeted at small and privately owned CWS, which are underrepresented in past California water rate surveys. We combined information on water rate structures from three secondary data sources – (i) American Water Works Association Water Rate Surveys, (ii) Black & Veatch California Water Charge Surveys, (iii) California State Water Resources Control Board (SWRCB) Electronic Annual Reports. In addition, we conducted our own survey of a random sample of water systems to better represent small and privately-owned systems. A full description of secondary datasets and our survey is provided in SI Text S2.

We restrict the study sample to CWS, which are systems serving year-round populations of at least 25 people. Wholesalers are excluded; we only include systems with retail residential customers since the analysis focuses on residential rates. The study period is 2006-2015 since a significant break point in PCWR adoption exists in 2006, as indicated by a Supremum Wald test. This timeframe also captures a period of rapid PCWR uptake; in 2006, about 44% of California utilities had PCWRs, which grew to 71% by 2015.

Our balanced panel represents an improvement over studies that rely on self-reported surveys from a single source. Self-reported rate structures might not reflect true water rates. Utility staff voluntarily respond to rate surveys from consultants and might not be familiar with how survey administrators define rate categories. We observed numerous inconsistencies in the raw data from secondary sources, including incorrectly reported rate types. To address these issues and improve data quality, we cross-checked reported rates across multiple secondary sources. In cases where cross-checking did not resolve the issue, we obtained additional information via communication with utility officials or from city council documents, including water rate studies, rate structures posted online, and city council resolutions.

The panel dataset includes water system characteristics and weather variables. Unmetered service connections and imported water information was compiled from Urban Water Management Plans filed with the SWRCB. Number of service connections and system ownership were obtained from the EPA Safe Drinking Water Information System; these variables are time invariant. Median household income for each water system service area was calculated using U.S. Census data and methods described in SI Text S2. County-level temperature was calculated based on data obtained from the NOAA National Centers for Environmental Prediction.

Based on these data, we create the following covariates. A full description is provided in SI Text S2. Capacity factors include utility size, household income, and extent of unmetered connections. Utility governance is classified based on system ownership. Ownership of CWS is categorized as private or public. Public ownership is further separated into special district and general-purpose government. Indicators for private ownership and special district are included in the regression models, with general-purpose government serving as the comparison category. Our water import variable, South-of-Delta water importer, indicates whether a utility purchases imported water and is located south of the Sacramento-San Joaquin Delta. These utilities hold junior water rights and likely face reductions in water allocations during drought periods. Finally, we capture peer effects by calculating the portion of other utilities that have adopted PCWRs in a given region. Number of customer complaints serve as a proxy of how engaged a community is regarding the quality of water service.

## Methods

We assess factors associated with PCWR adoption using logistic regression models.^2^ Our balanced panel dataset of 323 water systems captures a rapid period of adoption and has observations in survey years 2006, 2007, 2009, and 2011-2015.

The likelihood of a utility having a PCWR in a given year is modeled as:

15 $$\mathrm{Pr}({\mathrm{y}}_{\mathrm{it}}=1|\mathrm{X} )=f({\upbeta }_{0}+{\upbeta }_{\mathrm{it}}{\mathrm{C}}_{\mathrm{it}}+{\upgamma }_{\mathrm{jt}}{\mathrm{M}}_{\mathrm{jt}}+{\upeta }_{\mathrm{jt}}{\mathrm{R}}_{\mathrm{jt}}+{\propto }_{\mathrm{t}}{\mathrm{T}}_{\mathrm{t}})$$

where *y*_*it*_ is a binary indicator of a PCWR at utility *i* in year *t*. The probability of a utility having a PCWR is estimated as a function of capacity (*C*_*it*_), motivating (*M*_*it*_), and external factors (*R*_*jt*_). Capacity factors include utility size (number of service connections), median household income, and percent unmetered connections. Motivating factors include utility governance, South-of-Delta water importer, customer complaints, and peer effects. Annual maximum temperature is an external factor (*R*_*jt*_). The model includes year fixed effects (*T*_*t*_), which control for changes overtime such as statewide policy.

Results are reported as average marginal effects, which provide an interpretable estimate of the effect of each covariate on the likelihood of a utility adopting a PCWR. To calculate average marginal effects, we compare two hypothetical populations. For each utility-year observation, we calculate the probabilities for the case where the observation has the characteristic of interest (e.g. large utility = 1) and for the case where this characteristic is not present (e.g. large utility = 0). The difference in these two probabilities is the marginal effect for that single observation. The average marginal effect is the mean of all marginal effects across all observations.

## Results

### Summary Statistics: The Diffusion Process

**Fig. 2:**
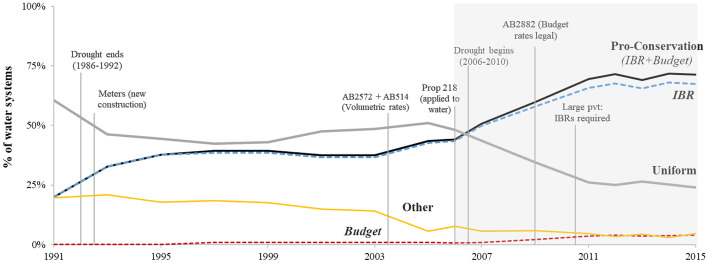
Water Rate Structures in California, 1991-2015. Note: The graph reports the share of utilities with each rate structure, using data from repeated cross-sections of water systems. Within each year, the portion of CWS with each water rate category is calculated. The four categories are: uniform, IBR, water budget, and other. The ‘other’ category is comprised mostly of flat rates (non-metered) as well as declining block rates and other rates that are not specified by the respondent. The gray area denotes our study period, years 2006-2015. In total, 578 utilities report in at least one year; the number of systems reported in a given year varies from 220 (in 1991) to 428 (in 2006)

Adoption of PCWRs, such as IBRs, appears to follow an S-curve (Fig. 2); this figure presents the share of utilities in each year with a given rate structure from 1991 to 2015. We find a dramatic shift towards PCWRs, which 71% of California utilities had by 2015, compared to half of the utilities in 2007 and only 20% in 1991. Our study period, 2006-2015, represents a rapid period of uptake. During this time, PCWRs became the most prevalent rate structure in California; by 2007, over half of community water systems had a PCWR. A transition away from uniform rates, towards IBRs and water budget rates, occurred during our study period. In recent years, PCWR adoption has plateaued, perhaps reaching saturation.

We estimate the adopter function (Eq. 2) with a fractional response model (Papke and Wooldridge 1996), which we fit using a logistic function and assuming $\overline{N }$=1. We find that annual average uptake of PCWRs is 3.0% (Table S1) during our study period, which is much faster than years 1991-2015 overall, which have an annual adoption rate of 2.0% (SI Text S5, Table S1).

### Summary Statistics: PCWR Adoption

In the 2006-2015 panel, most of the 323 CWS (74%) adopted PCWRs by year 2015. Coastal and southern regions of California had the highest prevalence of PCWRs in 2015, while the Sacramento River region had the lowest prevalence (Fig. S2). Utilities that never adopted PCWRs during this period are significantly smaller, serve lower income service areas, are less likely to be South-of-Delta importers, and have a lower portion of neighbors with PCWRs (Tables S2 and S3).

The portion of peers with a PCWR has a mean value of 67%, and varies widely across the sample, from 31 to 92% (Table 1). A substantial portion of utilities are junior water rights holders that rely on purchased water – 37% of utilities are South-of-Delta water importers. Among utilities with PCWRs, 46% are special districts, while only 13% are privately operated. In contrast, special districts are less common (39%) among utilities without PCWRs, while private operation is more prevalent (18%). Higher income levels are present in utility-year observations with PCWRs (mean: $73,594), compared to observations without PCWRs (mean: $64,415) (Table 1).

**Table 1:** Summary Statistics: 2006-2015 Panel

Variable	Definition	Mean	SD	Min	Max
Pro-conservation water rate, dummy variable	Indicator of utility with a PCWR in a given year	0.67	0.47	0	1
*Capacity*					
# Service connections, 2005	# of people served by utility in year 2005	16,046	29,776	64	380,450
% Unmetered service connections	Percent of service connections not metered	0.04	0.15	0	0.99
Median household income	Median household income in system service area, in $2017	70,529	22,962	27,144	195,552
*Motivation*					
Governance: Special district	=1 if special district	0.43	0.50	0	1
Governance: Private owner	=1 if privately owned utility	0.15	0.36	0	1
Governance: General-purpose government	=1 if general purpose government	0.42	0.49	0	1
South-of-Delta water importer	=1 if system is a junior rightsholder (i.e. located south of the Sacramento-San Joaquin Delta) and purchases imported water	0.37	0.48	0	1
# Customer complaints per connection	# of customer complaints per connection regarding service quality for years 2013-2015	0.004	0.01	0	0.11
% Neighbors with PCWR	Percent of other utilities with a PCWR in a given year, located in same hydrologic region	0.67	0.16	0.31	0.92
*External*					
Avg. annual max. temperature, °C	Average annual maximum temperature, in °C	34.1	5.3	17.1	45.6
	N	2,584			
	# water systems	323			

### Regression Results: PCWR Adoption

We assess factors associated with observing a PCWR in a given year. Results are presented in terms of marginal effects (Table 2). Observing a PCWR is found to be associated with both capacity and motivating factors. Utilities with PCWRs have greater capacity, as indicated by number of service connections, metering, and customer income (Table 2, Models 1-4). A percentage point increase in service connections is associated with more than a 2.4 percentage point increase in likelihood of having a PCWR (Table 2, Model 2). Utilities serving with larger service populations can take advantage of economies of scale that exist in the water sector. In addition, customer income levels affect the revenue base of a utility and the extent to which affordability concerns influence proposed rate changes.

**Table 2:** Regression Results, Marginal Effects

		(1)			(2)			(3)			(4)	
	ME		Std Err.	ME		Std Err.	ME		Std Err.	ME		Std Err.
*Capacity*												
ln(# service connections), 2005	0.027	***	0.007	0.024	***	0.007	0.031	***	0.007	0.027	***	0.007
% Unmetered service connections	-0.546	***	0.069	-0.509	***	0.070	-0.577	***	0.070	-0.514	***	0.070
ln(Median household income)	0.223	***	0.029	0.196	***	0.031						
*Motivation*												
Governance: Special district	0.050	**	0.020	0.051	***	0.020	0.059	***	0.020	0.060	***	0.020
Governance: Private owner	0.011		0.026	0.008		0.027	-0.002		0.027	-0.003		0.027
South-of-Delta water importer							0.064	***	0.020	0.043	**	0.020
# Customer complaints per connection	3.387	***	0.929	3.192	***	0.926	3.477	***	0.949	3.159	***	0.940
% Neighbors with PCWR^a^				0.204	***	0.077				0.326	***	0.075
*External*												
Avg. annual max. temperature, °C	0.0003		0.002	0.001		0.002	-0.002		0.002	0.000		0.002
Year fixed effects		Yes			Yes			Yes			Yes	
Log likelihood		-1,477			-1,473			-1,500			-1,490	
LR χ^2^		338			345			293			311	
Prob> χ^2^		0.000			0.000			0.000			0.000	
McFadden's R^2^		0.103			0.105			0.089			0.095	
N		2,584			2,584			2,584			2,584	
# Community Water Systems		323			323			323			323	

Delta method standard errors are reported for marginal effects

The dependent variable in all models is an indicator for having a PCWR; the unit of analysis is the utility-year

Delta method standard errors are reported

** Statistically significant at the 5% level; *** Statistically significant at the 1% level

^a^Represents *% Neighbors with PCWR* in each of the eight hydrologic regions in a given year

Significant motivating factors include special district governance, greater customer engagement, and peer effects. Special districts are associated with a 5.1 percentage point increase in likelihood of having a PCWR (Table 2, Model 2). A potential explanation for greater uptake of PCWRs among special districts is that this specialized governance structure allows customers to directly choose elected officials and voice their preferences on water pricing. Special districts can be more responsive to majority opinion, compared to private utilities and general governments. Majority opinion likely favors IBRs due to the right skew of residential water demand; adoption of IBRs will shift a greater portion of service cost to relatively few large water users.

More engaged customers can also enable adoption of PCWRs. Engagement of the community measured through customer complaints related to service quality; we find that utilities with a greater rate of customer complaints unrelated to pricing.

Water imports and junior water rights also influence uptake. South-of-Delta water importers have a similar magnitude for estimated marginal effect (Table 2, Model 3) as our special district indicator. When peer effects are also considered (Table 2, Model 4), the estimated marginal effect is smaller and less significant; this might be due to clustering of South-of-Delta utilities in southern hydrologic regions.

Having neighboring utilities with PCWRs is significant for explaining the adoption of pro-conservation rate structures. A utility is more likely to switch to a PCWR as the portion of their neighbors with PCWRs increases. In California, water rate cases often feature comparisons of rates at nearby utilities. This suggests that utilities compare and imitate water rate structures of neighbors.

The one external factor considered, average annual maximum temperature, is not significantly associated with the likelihood of PCWR adoption. This might be partly explained by county-year level weather data and arid conditions existing in much of California. Less variation in temperature exists compared to a study that also includes regions with more humid climates.

These findings are in agreement with supplemental analysis that uses a 1991-2015 panel dataset of 285 utilities (SI Text S4, Tables S4 and S5). We place greater emphasis on results from the 2006-2015 panel, since the 1991-2015 sample is dataset is over-representative of utilities that are larger, publicly-owned, and more reliant on water imports (Tables S6 and S7). Early adoption during years 1991-1995 is associated with metered connections and neighbor uptake of PCWRs (Table S8). When excluding peer effects, income is significantly associated with early adoption (Table S9, Model A5). In addition, South-of-Delta water importers have a greater likelihood of early adoption, although the estimated marginal effect is only significant at the 10% level (Table S10, Model A9).

## Conclusions and Policy Implications

This study focuses on understanding what motivates water utilities to adopt pro-conservation water rates. We find a dramatic shift away from uniform rates towards PCWRs, which 71% of California utilities had by 2015, compared to less than half of the utilities in 2006.

Results also indicate that PCWRs are more likely among utilities with greater capacity and stronger motivation to adopt. Capacity factors associated with adoption include size of service population and customer income level. Larger utilities can spread fixed costs over a more extensive customer base, including the costs of transitioning to a new rate structure. In contrast, systems serving lower-income communities might be less able to develop a rate case and face greater concerns regarding affordability. Significant motivating factors include peer adoption, greater customer engagement, special district governance, and junior rightsholders reliant on water imports. Growing adoption of PCWRs by peer utilities might facilitate learning or the establishment of new norms regarding rate structures. We find that a utility is more likely to adopt as the portion of their neighbors with PCWRs increases. Special districts might allow constituents to directly convey their preferences on water pricing, separate from other issues. In contrast, elections for officials of general-purpose governments feature a variety of issues beyond water services. And privately-operated utilities might not have officials elected by customers or might limit voting to property owners. Beyond governance structures and elections, the level of customer engagement as measured by recorded customer complaints is associated with a greater likelihood of PCWR adoption. Greater interaction between a utility and its customers might allow knowledge sharing regarding the impacts of a transition to PCWRs. Lastly, utilities with both junior water rights and reliance on water imports might be motivated to conserve as deliveries become more uncertain and/or costly during dry periods.

Barriers for adoption of PCWRs include systems with a larger portion of unmetered connections and small service populations. Technical and/or financial assistance might be required to design and implement a PCWR. Overall, this study provides insight into barriers to conservation pricing, which can inform policies to enable transitions and advance conservation goals.

It is unknown how PCWRs might evolve in the future, especially water budget rate structure. With new conservation targets set for all water utilities in California, water budget rates might be on the threshold of a rapid upward trajectory in uptake. Yet, it is also possible that this rate structure might not spread further because of its administration costs. These costs include public engagement to inform customers of how water budget rates will affect their billing.

In addition, utilities across the state are grabbling with the reality that per capita residential water use has had a downward trajectory since 2013 (Lee et al. 2021), yet fixed costs of water infrastructure need to be recovered, likely through increased volumetric prices in the future. Additional conservation gains would likely mean reduced revenues for water systems. A challenge is to enable utilities to encourage conservation, while ensuring that customers are rewarded with lower bills and utilities do not suffer revenue losses. To further encourage PCWRs, the state could expand revenue decoupling to publicly owned utilities. Decoupling is especially relevant as utilities cope with COVID-19 impacts, including reduced ability of customers to pay water bills and of utilities to sustain budgets (Eastman et al. 2020). Assistance measures being considered nationwide include deferred payments for customer bills and financial support for a portion of utility operational budgets (i.e. decoupling customer payments from water use). Expanded decoupling policy could offer utilities a way to ensure conservation goals, affordability, and reliable infrastructure.

State policies have allowed investor-owned utilities in California to decouple water sales from revenue since 2008, so that fixed costs can be recovered even if water use declines. Such policies are widely implemented in electricity and natural gas sector, but not for water utilities. At present, nearly half of U.S. states have adopted decoupling of sales from revenue for electricity and/or natural gas utilities (C2ES 2019). In California, all electricity utilities must have a decoupling plan and receive incentives for meeting efficiency goals. Decoupling has not widely spread to water utilities run by local governments. In addition, rate stabilization mechanisms could be developed in order to reduce financial risks due to demand swings. This is particularly critical as more severe and prolonged drought periods are expected in the future.

## Supplementary Information

Below is the link to the electronic supplementary material.

Supplementary file1 (DOCX 254 KB)

## Data Availability

Data associated with this research is available at Dataverse (https://dataverse.harvard.edu/dataverse/allaire).

## Footnotes

1 Key differences between general and special purpose governments are service area boundaries and debt limits. Special districts can more easily annex new service areas since their boundaries do not need to follow municipal borders. In addition, special districts typically do not face debt limits when issuing revenue bonds, which is an attractive financing option for developers (Foster 1997).

2 In supplemental analysis, we assess factors that influence timing of adoption. See SI Text S3, S6, S7 for a description and results of first-wave vs second-wave PCWR adoption.

## References

[CR1] Arbués F, García-Valiñas MÁ, Martínez-Espiñeira R (2003) Estimation of residential water demand: a state-of-the-art review. J Soc Econ 32(1):81–102. 10.1016/S1053-5357(03)00005-2

[CR2] Berry FS, Berry WD (2018) Innovation and diffusion models in policy research. In Theories of the Policy Process (Weible, C. and P. Sabatier (Eds.)). West View Press

[CR3] Boyer CN, Adams DC, Borisova T (2014) Drivers of price and nonprice water conservation by urban and rural water utilities: an application of predictive models to four southern states. J Agric Appl Econ 46(1):41–56. 10.1017/S1074070800000626

[CR4] Boyer CN, Adams DC, Borisova T, Clark CD (2012) Factors driving water utility rate structure choice: evidence from four southern U.S. states. Water Resour Manag 26(10):2747–2760. 10.1007/s11269-012-0043-z

[CR5] Brandes O, Renzetti S, Stinchcombe K (2010) Worth every penny: a primer on conservation-oriented water pricing. University of Victoria POLIS Project on Ecological Governance. http://poliswaterproject.org/sites/default/files/Pricing%20Primer%20Final.pdf

[CR6] Brent DA, Ward MB (2019) Price perceptions in water demand. J Environ Econ Manag 98:102266. 10.1016/j.jeem.2019.102266

[CR7] C2ES (2019) Decoupling policies. Center for Climate and Energy Solutions. Arlington, VA. http://www.c2es.org/document/decoupling-policies/

[CR8] Chestnutt T, Beecher J, Mann P, Clark D, Hanemann WM, Raftelis G, McSpadden C, Pekelney D, Christianson J, Krop R (1997) Designing, evaluating, and implementing conservation rate structures. California Urban Water Conservation Council

[CR9] Cosgrove WJ, Loucks DP (2015) Water management: current and future challenges and research directions. Water Resour Res 51(6):4823–4839. 10.1002/2014WR016869

[CR10] Dalhuisen JM, Florax RJGM, de Groot HLF, Nijkamp P (2003) Price and income elasticities of residential water demand: a meta-analysis. Land Econ 79(2):292–308. 10.2307/3146872

[CR11] Dearing JW, Cox JG (2018) Diffusion of innovations theory, principles, and practice. Health Aff (Project Hope) 37(2):183–190. 10.1377/hlthaff.2017.1104 29401011 10.1377/hlthaff.2017.1104

[CR12] Dinar A, Ash T (2015) Water budget rate structure: experiences from several urban utilities in southern California. In Lago M., Mysiak J., Gómez C., Delacámara G., Maziotis A. (eds) Use of Economic Instruments in Water Policy (Vol. 14, pp. 147–170). Springer.

[CR13] Eastman L, Smull E, Patterson L, Doyle M (2020) COVID-19 Impacts on water utility consumption and revenues: preliminary results. Raftelis and Duke University. https://nicholasinstitute.duke.edu/sites/default/files/publications/COVID-19-Resources-Impacts-on-Water-Utility-Consumption-and-Revenues.pdf

[CR14] Foster K (1997) The political economy of special purpose government. Georgetown University Press

[CR15] Gaur S, Diagne M (2017) California water rate trends: maintaining affordable rates in a volatile environment. J Am Water Works Assoc 109:46–52. 10.5942/jawwa.2017.109.0127

[CR17] Gurung A, Martínez-Espiñeira R (2019) Determinants of the water rate structure choice by Canadian municipalities. Util Policy 58:89–101. 10.1016/j.jup.2019.04.003

[CR18] Hanak E (2005) Water for growth: California’s new frontier. Public Policy Institute of California

[CR19] Hewitt J (2000) An investigation into the reasons why water utilities choose particular residential rate structures. Oxford University Press for the Waorld Bank. In The Political Economy of Water Pricing Reforms

[CR20] Ito K (2014) Do consumers respond to marginal or average price? evidence from nonlinear electricity pricing. Am Econ Rev 104(2):537–563. 10.1257/aer.104.2.537

[CR21] Kenney DS, Goemans C, Klein R, Lowrey J, Reidy K (2008) Residential water demand management: lessons from Aurora, Colorado. JAWRA J Am Water Resour Assoc 44(1):192–207. 10.1111/j.1752-1688.2007.00147.x

[CR22] Kwon S-W, Bailey DB (2019) Examining the variation in local water sustainability practices. Soc Sci J 56(1):107–117. 10.1016/j.soscij.2018.08.011

[CR23] Lee J, Nemati M, Dinar A (2021) Historical trends of residential water use in California: effects of droughts and conservation policies. Appl Econ Perspect Policy Early View. 10.1002/aepp.13149

[CR24] Montginoul M (2007) Analysing the diversity of water pricing structures: the case of France. Water Resour Manag 21(5):861–871. 10.1007/s11269-006-9104-5

[CR25] Mullin M (2008) The conditional effect of specialized governance on public policy. Am J Polit Sci 52(1):125–141. 10.1111/j.1540-5907.2007.00303.x

[CR26] Olmstead S, Hanemann M, Stavins R (2007) Water demand under alternative price structures. J Environ Econ Manag 54(2):181–198. 10.1016/j.jeem.2007.03.002

[CR27] Ostrom V, Tiebout C, Warren R (1961) The organization of government in metropolitan areas: a theoretical inquiry. Ame Polit Sci Rev 55(4):831–842

[CR28] Papke L, Wooldridge J (1996) Econometric methods for fractional response variables with an application to 401(k) plan participation rates. J Appl Econom 11(6):619–632

[CR29] Pinto SF, Marques CR (2015) Tariff Structures for water and sanitation urban households: a primer. Water Policy wp2015188. 10.2166/wp.2015.188

[CR31] Renzetti S, Brandes OM, Dupont DP, MacIntyre-Morris T, Stinchcombe K (2015) Using demand elasticity as an alternative approach to modelling future community water demand under a conservation-oriented pricing system: an exploratory investigation. Can Water Resour J 40(1):62–70. 10.1080/07011784.2014.985508

[CR32] Reynaud A, Renzetti S, Villeneuve M (2005) Residential water demand with endogenous pricing: the Canadian case. Water Resour Res 41(11). 10.1029/2005WR004195

[CR33] Rogers E (2003) Diffusion of innovations (Fifth). Free Press

[CR34] Teodoro MP (2010) Contingent professionalism: bureaucratic mobility and the adoption of water conservation rates. J Public Adm Res Theor 20(2):437–459. 10.1093/jopart/mup012

[CR35] Wichman CJ (2014) Perceived price in residential water demand: evidence from a natural experiment. J Econ Behav Organ 107:308–323. 10.1016/j.jebo.2014.02.017

