# Supplementary material for: What Drives Water Utility Selection of Pricing Methods? Evidence from California
Source: Water Resour Manag (Dordr). 2021 Nov 4;36(1):153–69. doi: 10.1007/s11269-021-03018-8 (PMC8566021; doi:10.1007/s11269-021-03018-8)
Supplement: Supplementary file 1 — Supplementary file1 (DOCX 254 KB) [file 11269_2021_3018_MOESM1_ESM.docx]

*Water Resources Management*

Supplementary Information for

**What Drives Water Utility Selection of Pricing Methods? Evidence from California**

M. Allaire^1^ and A. Dinar^2^

^1^Department of Urban Planning and Public Policy, University of California, Irvine.

^2^School of Public Policy, University of California, Riverside.

**Contents of this file**

Supplementary Text S1 to S6

Figures S1 to S2

Tables S1 to S10

**Introduction**

This Supplementary Information provides text, figures, and tables of summary statistics and regression results that are supplementary to the article.

**Supplementary Text**

**S1. Pro-Conservation Water Rates and Use in California**

**S1.1 Transitions in California Water Rates**

In recent years, several California utilities have shifted their water pricing schemes to encourage conservation, while collecting adequate revenue. Identifying factors that facilitate transitions to PCWR is policy-relevant as California overhauls its approach to conservation and mandates water use targets for all urban water systems.

Many water managers across California must consider how to cope with increasing hydrologic uncertainty. In the face of increased drought frequency and severity as well as growing urban populations, securing water for communities will likely require a mix of supply augmentation and demand-side management. One of the most severe California droughts in modern history ended in 2017. During this event, an order was issued by the Governor’s Office for all municipalities to reduce water use by 25%. While many utilities achieved these reductions, by mid-2018, water use in many locations had rebounded to pre-drought levels after restrictions were lifted. Many conservation measures had temporary effects; achieving long-term reductions might require alternative strategies (CDWR and SWRCB, 2018).

Since the historic drought, California is revising its approach to water conservation. The state will no longer issue mandates for all utilities to achieve the same percent reductions in usage. Such uniform orders do not account for differences in past conservation achievements and customer characteristics. Two statewide water conservation bills, passed in 2018, aim to make conservation a “way of life”. This new legislation aims to reduce per capita use by requiring utilities to meet targets for indoor and outdoor use. Utilities have flexibility in deciding how to meet these targets.

Water pricing is generally considered to be more economically efficient than mandates for encouraging conservation (Olmstead and Stavins 2009). To achieve adequate reductions in per capita use, prices would need to reflect the value of water for its next best use. However, rate regulations in California and in many other states prevent scarcity pricing from being implemented. In California, water utilities cannot charge rates above the cost of service, as established in Proposition 218. Therefore, utilities cannot simply raise water rates to achieve conservation. Furthermore, there is evidence that water utility managers perceive customer demand as unresponsive to price changes (Griffin 2001; Olmstead et al. 2007).

Given these restrictions on conservation pricing, how can water rates be designed to motivate conservation? Rather than simply raising the price of water within the current rate structure, utilities may need to overhaul the rate structure itself. Revised structures might spur customers to be more aware of the water rates they face; this in turn could also make their own usage more salient. Many residential consumers are unaware of the prices charged for their water and electricity (Bell & Griffin, 2008; Gaudin, 2006; Jessoe & Rapson, 2014). A utility’s choice of rate structure could influence customer awareness (Kenney et al., 2008; WRA, 2003). For example, an IBR might increase saliency, more than a uniform rate, because of the possibility that customers receive a high water bill if their use crosses into higher tiers.

**S1.2 Adoption Drivers in California**

In this study, we aim to identify drivers of PCWR structure transitions in California. External factors examined include drought conditions and state-level policy. In California, two droughts coincided with the transition to PCWRs – the 1986-1992 and 2006-2010 droughts. The former was one of the longest in state history, while the later was the first to have a statewide emergency declaration. This later drought event led to considerable reductions in water imports, delivered through the state water project; long-term restrictions were codified in 2009 through the Delta Reform Act. Many utilities in California receive a portion of their supply from water imports. Reductions in imports particularly affected junior water rights-holders, which comprise utilities south of the Bay Delta. Under prior appropriation doctrine, senior rights-holders receive their allocations first. Therefore, junior rights-holders (South-of-Delta importers) face uncertainty regarding how much of their allocation will be received. Drought conditions and concerns over water imports could motivate utilities to revise rate structures in order to achieve conservation.

In addition, a variety of water conservation and water rate policies might influence choice of rate structure. Utilities must comply with Proposition 218, which prohibits revenues from exceeding cost of service (ACWA, 2007). In 2006, this proposition was formally acknowledged as applying to water service. Rate structures must be designed to avoid surplus revenue. This policy might motivate some utilities to shift to PCWRs, such as increasing block rates, because utilities can justify greater costs of additional water sources, such as purchased water or developing new sources. More generally, the possibility of legal action and costs of developing a rate case will influence choice of rate structure.

State mandates for metering and volumetric pricing have influenced utilities for the past several decades. Since 1992, meters have been required by Senate Bill SB 229 for new construction within service areas with 3,000 or more customers. Those purchasing water from the Central Valley Project were required to charge volumetric rates by 2010, as part of Assembly Bill AB 514 passed in 2003. In 2004, the state required meters on all existing connections by 2025 and billing based on volumetric rates by 2010, as part of AB 2572. Universal metering was not achieved during our study period and was actively opposed by some California communities. For example, prior to the 2004 statewide mandate, Fresno and Sacramento residents voted to maintain local bans on metering.

Private utilities, more recently, have been impacted by state mandates. Required volumetric rates were passed for investor-owned utilities in 2009, through AB 975. And, in 2010, large investor-owned utilities were required to have increasing block rates (CPUC, 2010). Transitions to PCWRs by private utilities were also facilitated by state policies to decouple water sales from revenue, so that fixed costs can be recovered even if water use declines (CPUC, 2005). As of 2008, investor-owned utilities can reconcile differences between revenue and sales each year (CPUC, 2017).

Statewide policy also emphasizes conservation targets, which were established under the 20×2020 Water Conservation Plan. The goal was to reduce California per capita water use in urban areas by 20% by 2020 (CDWR, 2010). PCWRs are one strategy for reducing per capita usage.

A specific type of PCWR, water budget rates, might have expanded in use after state legislation in early 2009 that clarified that budget rates are legal (AB 2882). This policy permits utilities to penalize wasteful water use, and thus recover costs of securing additional supply and conservation activities. Yet, a barrier to budget rates might be greater regulatory scrutiny for utilities that adopt it (Baerenklau et al. 2014).

**S2. Data: 2006-2015 Panel**

In the Main Text, the Data section (Section 5) provides a summary of the 2006-2015 panel; a more complete description is provided here. This panel includes 323 community water systems. Information on water rate structures was compiled from three secondary data sources – (i) American Water Works Association (AWWA) Water Rate Surveys, (ii) Black & Veatch (B&V) California Water Charge Surveys, (iii) California State Water Resources Control Board (SWRCB) Electronic Annual Reports (EAR). AWWA surveys are available for years 2005, 2007, 2009, 2011, 2013, and 2015. AWWA primarily reports rate structure type and average customer bill. Number of water systems reporting varies each year, from 153 in 2015 to 393 in 2011. B&V data are available from biennial surveys from 1991-2003 and 2006. B&V surveys report information on rate structure type, monthly charges, and average residential water use. Number of water systems reporting varies each year, from 220 in 1991 to 352 in 2003. SWRCB reports rates for all community water systems (CWS) in California from 2011-2015, we only retained CWS that reported rate structures in other datasets prior to 2011.

In addition, in order to capture underrepresented utilities (small systems and privately owned) in the secondary datasets, an email survey was conducted for a random sample. Therefore, our study does not only represent large, retail systems. Out of the 81 large, privately owned utilities in California, we selected 20 utilities, based on a simple random sample. For small CWS, we restricted our sample to those serving more than 500 people. Out of the 288 CWS in California with service populations between 500 and 10,000 people, we sampled 150 systems. We anticipated a response rate of 50%. For each sampled utility, we contacted the staff member who replied to the most recent SWRCB Electronic Annual Report. We requested residential rate structures from 2006-2015. Emails were first sent in May 2019, with up to three follow-up requests, and this survey was concluded in August 2019. We received complete information from 12 large private CWS (60% response rate) and 70 small CWS (47% response rate).

Based on these data, we create the following covariates. Capacity factors include utility size, household income, and extent of unmetered connections. Utility size is represented by the number of service connections in year 2005. Income data at the level of zip code tabulation areas (ZCTA) were obtained from the U.S. Census. We calculate median household income in each year at the water system level by using areal interpolation and calculate a population-weighted average of income at each system. Weights are based on (i) the portion of a ZCTA’s land area located with the service area boundary, and (ii) the total population of a zip code. Boundaries of ZCTAs (O’Neill, 2012) and water system service areas (Tracking California, 2019) were intersected in order to calculate the portion of each zip code located with a given service area. Unmetered connections as a portion of total service connections was calculated based on the number to unmetered and total service connections from the SWRCB.

Our water import variable, South-of-Delta water importer, indicates whether a utility purchases imported water and is located south of the Sacramento-San Joaquin Delta. This variable is equal to one if a system imported 1% or more of total water produced in 2005 and if the system is located in the following counties in central and southern California: Alameda, Alpine, Calaveras, Contra Costa, Fresno, Imperial, Inyo, Kern, Kings, Los Angeles, Madera, Mariposa, Merced, Monterey, Orange, Riverside, San Benito, San Bernardino, San Diego, Santa Barbara, Santa Clara, Santa Cruz, Stanislaus, Tulare, Tuolumne, Ventura.

Customer complaints per connection is equal to the total number of complaints for years 2013-2015, divided by number of service connections. Data are not available for years prior to 2013. Complaints are related to service quality (e.g. outages, pressure, perceived water quality, illness), not water rates. We consider this variable to be a proxy for level of customer engagement and pressure on a water utility.

In order to assess the association between climate conditions and choice of rate structure, we calculate average annual maximum temperature from the U.S. Climate Prediction Center (CPC) Gauge-Based Analysis of Global Daily Precipitation (NOAA, 2017). These gridded, daily climate data were converted to county-level by calculating the average values of cells within county boundaries. We do not include precipitation, as it is moderately correlated with water imports; to allow for clearer interpretation of coefficient estimates on South-of-Delta water importer, we consider temperature. In addition, local temperature is expected to be more closely associated with customer water use than precipitation. In California, most precipitation occurs within a few months, October to March. Parts of the state are semi-arid.

Finally, we capture peer effects by calculating the portion of other utilities that have adopted PCWRs in a given region. We divide the number of neighboring utilities with a PCWR (i.e. not including a utility’s own observation) by the total number of neighboring utilities. Our eight regions are based on hydrologic regions, designated by the California Department of Water Resources (CDWR, 2010) and State Water Resources Control Board, as shown in Figure S1. Hydrologic regions differ in terms of watershed, climate, and water use targets for the statewide water conservation plan, which requires a 20% state-level reduction by 2020 from 2005 levels of per capita water use. Reductions vary considerably by hydrologic region, ranging from a 17% to 30%.

**S3. Methods: PCWR Adoption 1991-2015**

Supplemental analysis examines the long-term evolution of water rate structures across 25 years in California. We assess the diffusion of PCWRs using a repeated cross-section of 578 utilities from 1991- 2015. In the main text, we present results from a 2006-2015 panel that is more representative of CWS in California overall and can consider several water system characteristics that are not available prior to 2005.

In the supplemental analysis, we identify two major waves of adoption. The first wave, 1991-1995, experienced the fastest annual adoption rate (4.4%) (Text S4, Table S1). We examine differences in adoption drivers between this first and second wave. The 1991-2015 dataset contains 285 CWS, which are a subset of utilities in the repeated cross-sectional data. We define the first wave as occurring from 1991-1995 and the second wave occurring between 1997-2015. Separate regression models are developed for first-wave adoption, second-wave adoption, and never adopting. The likelihood of a utility having a particular rate structure is modeled as:

Pr(y_i_=1|X )=Φ(β_0_ + β_i_ C_i_ + γ_j_ M_j_ + η_j_ R_j_) (S1)

where *y_i_* is a binary indicator of having a PWCR at utility *i*. The probability of a utility having a given rate is estimated as a function of capacity (*C_i_*), motivating (*M_i_*), and external factors (*R_j_*). Capacity factors include an indicator of large utility size, percent unmetered connections, and household income. Large utilities are defined as serving over 10,000 people (EPA, 2013)(EPA, 2013)(EPA, 2013). Motivating factors include governance structure, South-of-Delta water imports, and portion of neighbors that adopted a PCWR during the first wave. An external factor is average annual maximum temperature.

**S4. Data: Additional Study Samples**

Two additional samples are created to examine differences in adoption drivers between the first and second waves. The second sample we create is a repeated cross section, comprised of a total of 578 utilities, which is based on biennial surveys from 1991-2003, 2005-2007, 2009, and 2011-2015.

The third and final dataset created is a panel of 285 CWS, which are a subset of CWS in the repeated cross-section that report rate structures at least once during each of the two adoption waves: (i) first wave (1991-1995), and (ii) second wave (1997-2015)^[[1]](#footnote-1)^. The time break between the first and second wave was selected based on Figure 2 in the Main Text, which depicts rapid uptake of PCWRs during 1991-1995, which then plateaus.^[[2]](#footnote-2)^

While rate structure information is available from 1991 onward, several water system characteristics are not available prior to 2005, which is the first year SWRCB offers digital reports and datasets. As a result, we emphasize findings based on the 2006-2015 panel. In addition, utilities in this repeated cross-sectional sample are not representative of the 2,818 CWS in California as a whole – these utilities are larger and have more metering and public ownership (Table S6). When only comparing our 2006-2015 sample to California CWS serving 3,000 people or more, differences in service population and private ownership are no longer significant (Table S7). These differences are expected, since CWS with greater capacity are more likely to respond to water rate surveys. While our sample is not representative of smaller CWS with a large portion of unmetered connections, this is not expected to affect the generalizability of results since these smaller utilities serve a small portion of the state population.

**S5. Summary Statistics: The Diffusion Process, 1991-2015**

Adoption of PCWRs, such as IBRs, appears to follow an S-curve (Figure 2 in the Main Text). During years 1991 to 2015, we identify two major waves of adoption. The first wave of rapid transition from uniform rates to PCWRs occurred in the later years of the long-duration 1986-1992 drought. The early 1990s transition to PCWRs represents ‘early adopters,’ while those adopting up until 2006 can be considered the ‘early majority’ (Figure 1 in the Main Text). During this first wave, utilities mostly shift to IBRs, away from uniform rates. The portion of water systems with IBRs jumped from 20% in 1991 to 33% in 1993; at the same time, uniform rates declined from use in 60% of systems to 46%.

A second wave of PCWR adoption occurred from 1997-2015 and involved a transition away from flat and uniform rates. Between 2003 and 2005, flat rates dropped from being used by 12% of utilities to 3%. Most transitions took place from 2003-2011, which included both IBRs and water budget rates. By 2003, about half of the California population lived in a service area with IBRs, while only 7% had flat rates (Hanak 2005).

How quickly PCWR adoption reaches a ceiling, differs between the two waves. In the first wave, it took four years (from 1991 to 1995) to reach a ceiling of about 38%, which was maintained for eight years. In contrast, during the second wave, it took eight years (from 2003 to 2011) to arrive at a level of 70%, which has persisted, so far, for four years.

Across the full study period, 1991-2015, the annual average uptake of PCWRs is 2.0% (Table S1). Adoption is much faster during the first wave, which has an annual average growth of 4.4%, compared to the second wave. This is due to stagnant rate structures in the early part of the second wave. During the latter part of the second wave, 2006-2015, annual average growth is 3.0%.

**S6. Summary Statistics: 1991-2015 Panel**

Similar summary statistics are found in our panel samples from 2006-2015 (Table 1 in the Main Text) and 1991-2015 (Table S4). In the 1991-2015 panel, many CWS (41%) adopt PCWRs in the first-wave, while few never adopt (24%) (Table S4). By 2015, 76% of CWS in this panel of 285 systems have PCWRs. This observation is similar to our repeated cross-sectional sample, in which 71% of CWS in 2015 have a PCWR (Figure 2 in the Main Text). Utilities with PCWRs during the first-wave of adoption are more likely to be public utilities, have fewer unmetered connections, and more neighbors with PCWRs (Table S9). In contrast, utilities that never adopt PCWRs tend to have more unmetered connections, are less reliant on water purchases, and have fewer neighbors with PCWRs.

This dataset is over-representative of utilities that are larger and have greater water purchases and public ownership (Tables S6 and S7). As a result, we place greater emphasis on results from the 2006-2015 panel, which are presented in the Main Text.

**S7. Regression Results: 1991-2015 Panel**

Results for the 1991-2015 panel largely agree with findings based on our preferred 2006-2015 panel. For the 1991-2015 panel dataset of 285 CWS, we compare factors associated with waves of PCWR adoption. Utilities that never adopt PCWRs are less reliant on purchased water, are located in regions with fewer peers adopting PCWRs, and have less capacity, as indicated by greater portion of unmetered connections (Table S8, Model A4). Peer effects are significant during the first-wave of adoption; an increase in neighbors with PCWRs is associated with greater likelihood of adopting a PCWR (Table S8, Model A1). In addition, utilities in the first wave had greater capacity to transition to PCWRs; fewer unmetered connections are strongly associated with first-wave adoption.

Compared to utilities that never adopted PCWRs, early adopters are more reliant on purchased water. Being a South-of-Delta water importer is associated with first-wave adoptions; however, this estimated marginal effect is only significant at the 10% level (Table S9, Model A9). Second wave adoptions are not associated with being a South-of-Delta water importer. This may suggest that drought conditions and impacts on water deliveries served as motivating factors during the first wave. The first wave coincided with the end of a six-year drought (1987-1992); the driest period of this event was late 1990 to late 1991. Statewide reservoir storage declined by 1989 to 40% of average levels (Jones et al., 2015). This led to a drastic reduction in deliveries from the State Water Project, which in 1991 only provided 30% of requested urban deliveries (Jones et al., 2015).

During the second wave, utilities switching to PCWRs in this period were more likely to be privately owned, compared to the first wave (Table S5). Compared to CWS owned by municipal governments, privately owned have a 36.8 percentage point higher probability of adopting PCWRs during this second-wave (Table S8, Model A2). Prior to this second wave of adoption, private utilities were significantly less likely to adopt IBRs (Hanak 2005). Major changes in state policies regarding private utilities may have motivated some of these transitions. For example, California mandated volumetric rates for investor-owned utilities in 2009 and implemented decoupling programs.

**References**

ACWA. (2007). *Proposition 218. Local Agency Guidelines for Compliance*. Association of California Water Agencies.

Baerenklau, K. A., Schwabe, K. A., & Dinar, A. (2014). The Residential Water Demand Effect of Increasing Block Rate Water Budgets. *Land Economics*, *90*(4), 683–699. https://doi.org/10.3368/le.90.4.683

Bell, D. R., & Griffin, R. C. (2008). An annual quasidifference approach to water price elasticity. *Water Resources Research*, *44*(8). https://doi.org/10.1029/2007WR006233

CDWR. (2010). *20×2020 Water Conservation Plan*. California Department of Water Resources.

CDWR and SWRCB. (2018). *Making Water Conservation a California Way of Life* (p. 61). California Department of Water Resources (CDWR) and State Water Resources Control Board (SWRCB). https://water.ca.gov/-/media/DWR-Website/Web-Pages/Programs/Water-Use-And-Efficiency/Make-Water-Conservation-A-California-Way-of-Life/County-Drought-Planning/Files/Making-Water-Conservation-a-CA-Way-of-Life-Pimer.pdf

CPUC. (2005). *Water Action Plan 2005*. California Public Utilities Commission. https://www.cpuc.ca.gov/uploadedFiles/CPUC_Public_Website/Content/Utilities_and_Industries/Water/water_action_plan_final_12_27_05.pdf

CPUC. (2010). *Water Action Plan 2010*. California Public Utilities Commission. http://docs.cpuc.ca.gov/PUBLISHED/Graphics/125501.PDF

CPUC. (2017). *Water Rates Scenario Planning*. California Public Utilities Commission.

EPA. (2013). *Providing safe drinking water in America: 2013 National Public Water Systems compliance report* (EPA 305-R-15-001). Environmental Protection Agency.

Gaudin, S. (2006). Effect of price information on residential water demand. *Applied Economics*, *38*(4), 383–393. https://doi.org/10.1080/00036840500397499

Griffin, R. C. (2001). Effective Water Pricing. *JAWRA Journal of the American Water Resources Association*, *37*(5), 1335–1347. https://doi.org/10.1111/j.1752-1688.2001.tb03643.x

Hanak, E. (2005). *Water for Growth: California’s New Frontier*. Public Policy Institute of California.

Jessoe, K., & Rapson, D. (2014). Knowledge Is (Less) Power: Experimental Evidence from Residential Energy Use. *American Economic Review*, *104*(4), 1417–1438. https://doi.org/10.1257/aer.104.4.1417

Jones, J., Anderson, M., Chung, F., Islam, N., Juricich, R., Kofoid, J., Lek, J., Reyes, E., Roos, M., Smith, T., Suits, B., Tipton, E., & Zhou, Y. (2015). *California’s Most Significant Droughts: Comparing Historical and Recent Conditions*. California Department of Water Resources. https://water.ca.gov/LegacyFiles/waterconditions/docs/California_Signficant_Droughts_2015_small.pdf

Kenney, D. S., Goemans, C., Klein, R., Lowrey, J., & Reidy, K. (2008). Residential Water Demand Management: Lessons from Aurora, Colorado. *JAWRA Journal of the American Water Resources Association*, *44*(1), 192–207. https://doi.org/10.1111/j.1752-1688.2007.00147.x

NOAA. (2017). *CPC Gauge-Based Analysis of Global Daily Precipitation*. Climate Prediction Center. http://iridl.ldeo.columbia.edu/SOURCES/.NOAA/.NCEP/.CPC/.UNIFIED_PRCP/.GAUGE_BASED/.GLOBAL/.v1p0/

Olmstead, S., Hanemann, M., & Stavins, R. (2007). Water demand under alternative price structures. *Journal of Environmental Economics and Management*, *54*(2), 181–198. https://doi.org/10.1016/j.jeem.2007.03.002

Olmstead, S. M., & Stavins, R. N. (2009). Comparing price and nonprice approaches to urban water conservation. *Water Resources Research*, *45*(4). https://doi.org/10.1029/2008WR007227

O’Neill, M. (2012). *Census Zip Code Tabulation Areas, California, 2010 [Shapefile]*. California Office of Statewide Health Planning and Development. https://earthworks.stanford.edu/catalog/stanford-dc841dq9031

Tracking California. (2019). *Water Boundary Tool*. Public Health Institute. https://trackingcalifornia.org/water-systems/water-systems-landing

WRA. (2003). *SMART Water: A Comparative Study of Urban Water Use Efficiency Across the Southwest*. Western Resource Advocates. https://www.waterboards.ca.gov/waterrights/water_issues/programs/hearings/cachuma/comments_rdeir/pacific_institute/4otherreports/wra_ch3smartwater2003.pdf


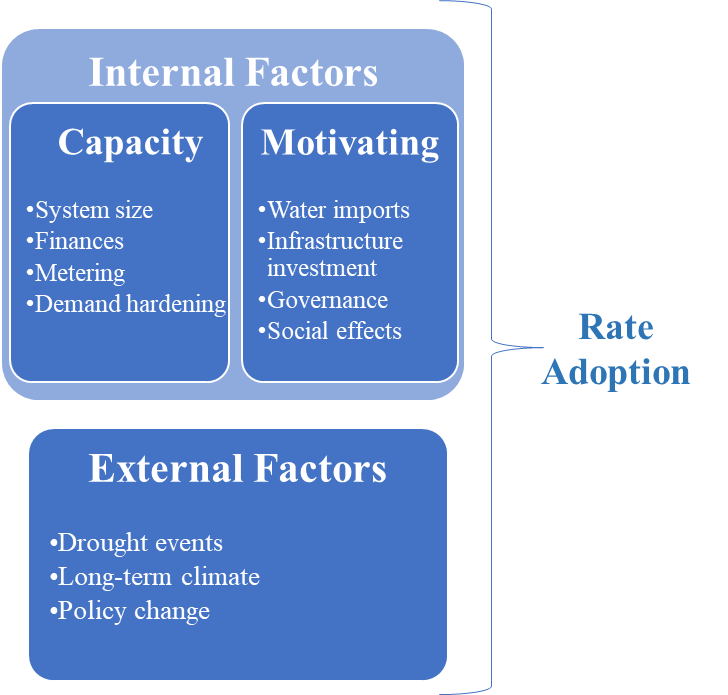


**Figure S1. Framework: Drivers of Utility Adoption of Pro-Conservation Rates**


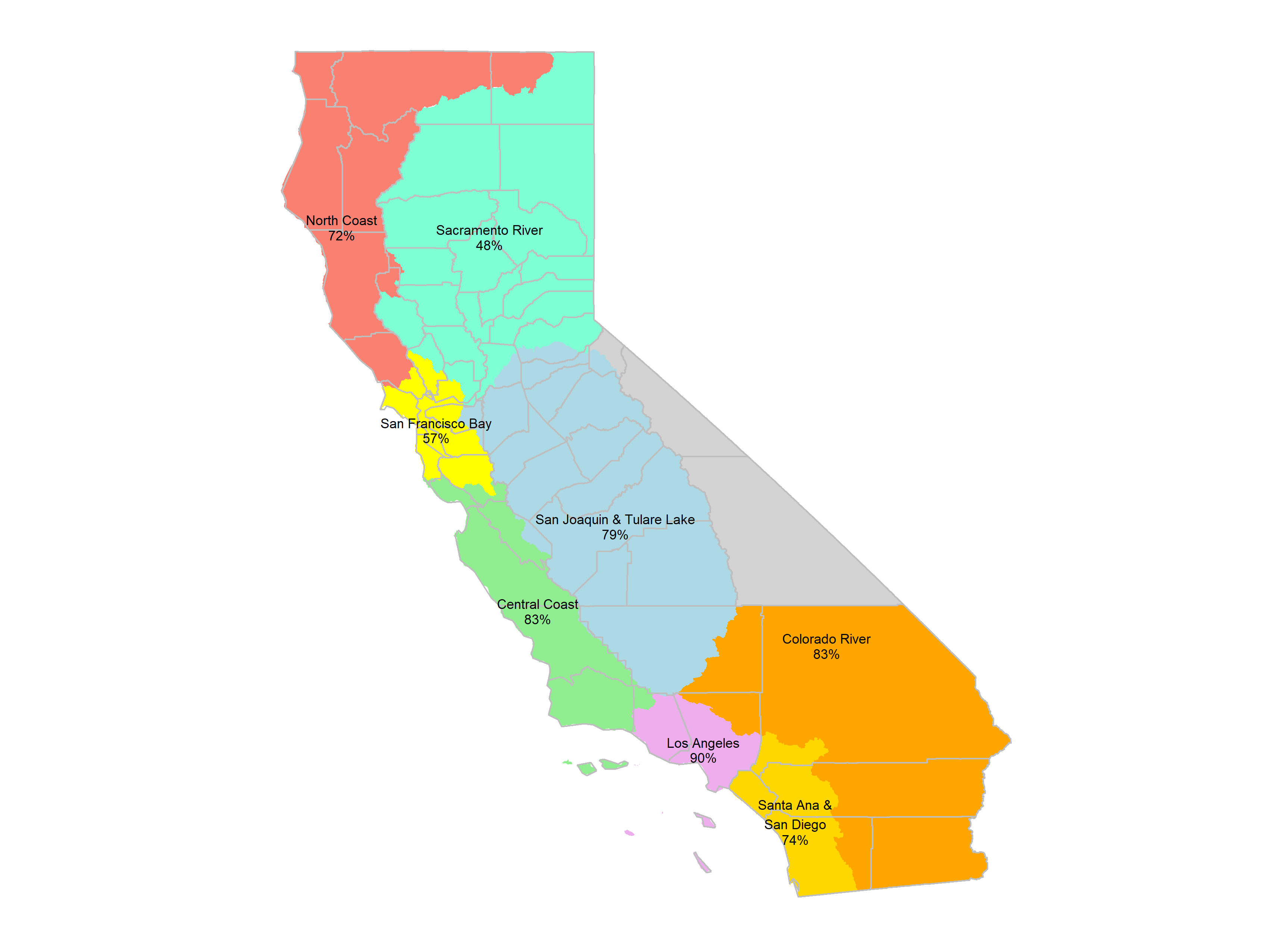


**Figure S2. Map of Study Regions.** This map presents the eight study regions and the share of utilities with a PCWR in 2015 for each region, based on the 2006-2015 panel sample. These regions are adapted from California’s ten hydrologic regions, designated by the California Department of Water Resources. Counties shaded in gray contain no study sample utilities.

List of counties included in each region: **North Coast** region (Del Norte, Humboldt, Mendocino, Sonoma, Siskiyou, Trinity); **Sacramento River** region (Butte, Colusa, El Dorado, Glenn, Lake, western parts of Lassen, Modoc, Nevada, Placer, western parts of Plumas, Sacramento, Shasta, Sierra, Sutter, Tehama, Yolo, Yuba); **San Francisco Bay** region (Alameda, Contra Costa, Marin, Napa, San Francisco, San Mateo, Santa Clara, Solano); **San Joaquin & Tulare Lake** region (Alpine, Amador, Calaveras, Fresno, Kern, Kings, Madera, Mariposa, Merced, Mono, San Joaquin, Stanislaus, Tulare, Tuolumne); **Central Coast** region (Monterey, San Benito, San Luis Obispo, Santa Barbara, Santa Cruz); **Los Angeles** region (Los Angeles, Ventura); **Santa Ana & San Diego** region (Orange, western parts of Riverside and San Diego, southwest parts of San Bernardino); and **Colorado River** region (Imperial, eastern parts of Riverside and San Bernardino).

*Note: Two DWR regions (North and South Lahontan) that contain few CWS are combined with other regions. The North Lahontan region contains four CWS that are assigned to the nearest neighboring region – three (in Lassen and Placer Counties) are assigned to the Sacramento River, while one (in Mono County) is assigned to the San Joaquin River and Tulare Lake region. The South Lahontan has ten CWS located in San Bernardino County that are assigned to the Colorado River region.*

*We also combine the San Joaquin River and Tulare Lake regions due to few sample CWS located in these regions – 19 and 20 CWS, respectively. Lastly, we split the South Coast region into two regions – Los Angeles and Santa Ana & San Diego – based on watershed boundaries, due to the large number of systems – 118 CWS.*

|  | **All years**:  1991-2015 | | | **First Wave**:  1991-1995 | | | **Second Wave**:  1997-2015 | | | ***Early Second Wave***:1997-2005 | | | ***Late Second Wave***: 2006-2015 | | |
| --- | --- | --- | --- | --- | --- | --- | --- | --- | --- | --- | --- | --- | --- | --- | --- |
|  | ME |  | Std Err. | ME |  | Std Err. | ME |  | Std Err. | ME |  | Std Err. | ME |  | Std Err. |
| Year | 0.020 | *** | 0.001 | 0.044 | *** | 0.007 | 0.023 | *** | 0.002 | 0.003 |  | 0.004 | 0.030 | *** | 0.004 |
| Log likelihood | -10.3 |  |  | -1.8 |  |  | -8.494 |  |  | -3.35 |  |  | -5.1 |  |  |
| LR χ^2^ | 128.3 |  |  | 38.13 |  |  | 66.11 |  |  | 0.72 |  |  | 58.95 |  |  |
| Prob> χ^2^ | 0.000 |  |  | 0.000 |  |  | 0.000 |  |  | 0.397 |  |  | 0.000 |  |  |
| McFadden's R^2^ | 0.069 |  |  | 0.021 |  |  | 0.053 |  |  | 0.000 |  |  | 0.029 |  |  |
| N | 16 |  |  | 3 |  |  | 13 |  |  | 5 |  |  | 8 |  |  |

Note: Table presents marginal effect estimates for $\emptyset$ in Eqn 2.

Delta method standard errors are reported for marginal effects.

* Statistically significant at the 10% level. ** Statistically significant at the 5% level. *** Statistically significant at the 1% level.

Table S1. Regression Results: Adopter Function.

|  |  | **With pro-conservation rate** | | | | **Without pro-conservation rate** | | | |
| --- | --- | --- | --- | --- | --- | --- | --- | --- | --- |
| Variable | Definition | Mean | SD | Min | Max | Mean | SD | Min | Max |
| *Capacity* |  |  |  |  |  |  |  |  |  |
| # Service connections, 2005 | # of people served by utility in year 2005 | 17,973 | 33,738 | 64 | 380,450 | 12,205 | 19,062 | 64 | 219,571 |
| % Unmetered service connections | Percent of service connections not metered | 0.01 | 0.07 | 0 | 0.85 | 0.09 | 0.23 | 0 | 0.99 |
| Median household income | Median household income in system service area, in $2017 | 73,594 | 23,484 | 28,617 | 195,552 | 64,415 | 20,574 | 27,144 | 140,771 |
| *Motivation* |  |  |  |  |  |  |  |  |  |
| Governance: Special district | =1 if special district | 0.46 | 0.50 | 0 | 1 | 0.39 | 0.49 | 0 | 1 |
| Governance: Private owner | =1 if privately owned utility | 0.13 | 0.34 | 0 | 1 | 0.18 | 0.39 | 0 | 1 |
| Governance: General-purpose government | =1 if general purpose government | 0.41 | 0.49 | 0 | 1 | 0.43 | 0.49 | 0 | 1 |
| South-of-Delta water importer | =1 if system is a junior rightsholder (i.e. located south of the Sacramento-San Joaquin Delta) and purchases imported water | 0.41 | 0.49 | 0 | 1 | 0.28 | 0.45 | 0 | 1 |
| # Customer complaints per connection | # of customer complaints per connection regarding service quality for years 2013-2015 | 0.005 | 0.01 | 0 | 0.11 | 0.003 | 0.01 | 0 | 0.04 |
| % Neighbors with PCWR | Percent of other utilities with a PCWR in a given year, located in same hydrologic region | 0.70 | 0.15 | 0.31 | 0.89 | 0.61 | 0.15 | 0.35 | 0.92 |
| *External* |  |  |  |  |  |  |  |  |  |
| Avg. annual max. temperature, °C | Average annual maximum temperature, in °C | 33.7 | 5.0 | 17.1 | 44.6 | 34.9 | 5.7 | 17.1 | 45.6 |
|  | N | 1,721 |  |  |  | 863 |  |  |  |
|  | # water systems | 245 |  |  |  | 164 |  |  |  |

Table S2. Summary Statistics: 2006-2015 Panel, by PCWR adoption

|  | **Full sample** | | | | **Ever had pro-conservation rate** | | | | **Never had pro-conservation rate** | | | | *Sig. Difference* |
| --- | --- | --- | --- | --- | --- | --- | --- | --- | --- | --- | --- | --- | --- |
|  | Mean | SD | Min | Max | Mean | SD | Min | Max | Mean | SD | Min | Max |  |
| *Capacity* |  |  |  |  |  |  |  |  |  |  |  |  |  |
| # Service connections, 2005 | 16,046 | 29,817 | 64 | 380,450 | 18,048 | 33,156 | 64 | 380,450 | 9,758 | 13,467 | 64 | 62,998 | ** |
| % Unmetered service connections, 2006 | 0.05 | 0.19 | 0 | 0.99 | 0.04 | 0.15 | 0 | 0.99 | 0.11 | 0.27 | 0 | 0.96 | *** |
| Median household income, 2006 | 71,730 | 23,241 | 29,630 | 189,596 | 74,342 | 23,622 | 30,604 | 189,596 | 63,523 | 20,012 | 29,630 | 140,771 | *** |
| *Motivation* |  |  |  |  |  |  |  |  |  |  |  |  |  |
| Governance: Special district | 0.43 | 0.50 | 0 | 1 | 0.45 | 0.50 | 0 | 1 | 0.38 | 0.49 | 0 | 1 |  |
| Governance: Private owner | 0.15 | 0.36 | 0 | 1 | 0.14 | 0.35 | 0 | 1 | 0.17 | 0.38 | 0 | 1 |  |
| Governance: General-purpose government | 0.42 | 0.49 | 0 | 1 | 0.41 | 0.49 | 0 | 1 | 0.45 | 0.50 | 0 | 1 |  |
| South-of-Delta water importer | 0.37 | 0.48 | 0 | 1 | 0.40 | 0.49 | 0 | 1 | 0.26 | 0.44 | 0 | 1 | ** |
| # Customer complaints per connection | 0.00 | 0.01 | 0 | 0.11 | 0.01 | 0.01 | 0 | 0.11 | 0.00 | 0.01 | 0 | 0.04 | * |
| % Neighbors with PCWR, 2006 | 0.51 | 0.12 | 0.31 | 0.74 | 0.52 | 0.12 | 0.31 | 0.74 | 0.47 | 0.11 | 0.35 | 0.74 | *** |
| *External* |  |  |  |  |  |  |  |  |  |  |  |  |  |
| Avg. annual max. temperature, °C, 2006 | 35.2 | 5.5 | 17.1 | 45.6 | 35.0 | 5.2 | 17.1 | 45.2 | 35.9 | 6.3 | 18.2 | 45.6 |  |
| N | 323 |  |  |  | 245 |  |  |  | 78 |  |  |  |  |

Note: Summary statistics in table above are provided for unique utilities; values in year 2006 are summarized for time variant variables. Table 1 in the Main Text provides summary statistics for all observations in the 2006-2015 panel dataset.

Table S3. Summary Statistics: Utilities in 2006-2015 Panel.

|  |  | **Full Sample** | | | |
| --- | --- | --- | --- | --- | --- |
| Variable | Definition | Mean | SD | Min | Max |
| First-wave Adoption | Indicator of utility adopting a PCWR during 1991-1995 | 0.41 | 0.49 | 0 | 1 |
| Second-wave Adoption | Indicator of utility adopting a PCWR during 1997-2015^1^ | 0.35 | 0.48 | 0 | 1 |
| Never Adopt | Indicator of utility not adopting a PCWR during 1991-2015 | 0.24 | 0.43 | 0 | 1 |
| *Capacity* |  |  |  |  |  |
| Large utility | Indicator of a utility serving over 10,000 people | 0.75 | 0.43 | 0 | 1 |
| % Unmetered service connections | Percent of service connections not metered during 2013-2015 | 0.05 | 0.20 | 0 | 1 |
| Median household income | Median household income in system service area, avg. value 2011-2015, in $2017 | 69,718 | 23,559 | 28,876 | 151,898 |
| *Motivation* |  |  |  |  |  |
| Governance: Special district | =1 if special district | 0.42 | 0.49 | 0 | 1 |
| Governance: Private owner | =1 if privately owned utility | 0.06 | 0.24 | 0 | 1 |
| Governance: General-purpose government | =1 if general purpose government | 0.53 | 0.50 | 0 | 1 |
| South-of-Delta water importer | =1 if system is a junior rightsholder (i.e. located south of the Sacramento-San Joaquin Delta) and purchases imported water | 0.34 | 0.47 | 0 | 1 |
| % Neighbors with PCWR | Percent of other utilities with a PCWR in the First-Wave (1991-1995), located in same hydrologic region | 0.41 | 0.16 | 0.14 | 0.72 |
| *External* |  |  |  |  |  |
| Avg. annual max. temperature 1986-1990, °C | Average annual maximum temperature, 1986-1990, in °C | 22 | 3.2 | 10.4 | 30 |
| Avg. annual max. temperature 1992-1996, °C | Average annual maximum temperature, 1992-1996, in °C | 21 | 3.2 | 9.5 | 30 |
| N |  | 285 |  |  |  |

Note: ^1^ Year 1996 was not a survey year for AWWA and B&V, so is unobserved for many utilities in the dataset.

Table S4. Summary Statistics: 1991-2015 Panel.

|  | **First-wave Adoption** | | | | **Second-wave Adoption** | | | | **Never Adopt** | | | | *Sig. Difference* | | |
| --- | --- | --- | --- | --- | --- | --- | --- | --- | --- | --- | --- | --- | --- | --- | --- |
| Variable | Mean | SD | Min | Max | Mean | SD | Min | Max | Mean | SD | Min | Max | *1st vs 2nd wave* | *1st vs Never* | *2nd vs Never* |
| *Capacity* |  |  |  |  |  |  |  |  |  |  |  |  |  |  |  |
| Large utility | 0.77 | 0.42 | 0 | 1 | 0.79 | 0.41 | 0 | 1 | 0.67 | 0.47 | 0 | 1 |  |  | * |
| % Unmetered service connections | 0.01 | 0.05 | 0 | 0.50 | 0.05 | 0.17 | 0 | 0.98 | 0.15 | 0.32 | 0 | 1 | ** | *** | *** |
| Median household income | 74,405 | 22,632 | 31,116 | 134,845 | 70,475 | 24,878 | 28,876 | 151,898 | 60,741 | 20,755 | 33,797 | 132,098 |  | *** | *** |
| *Motivation* |  |  |  |  |  |  |  |  |  |  |  |  |  |  |  |
| Special district | 0.44 | 0.50 | 0 | 1 | 0.40 | 0.49 | 0 | 1 | 0.42 | 0.50 | 0 | 1 |  |  |  |
| Private owner | 0.03 | 0.18 | 0 | 1 | 0.12 | 0.33 | 0 | 1 | 0.01 | 0.12 | 0 | 1 | ** |  | ** |
| General-purpose government | 0.54 | 0.50 | 0 | 1 | 0.49 | 0.50 | 0 | 1 | 0.57 | 0.50 | 0 | 1 |  |  |  |
| South-of-Delta importer | 0.42 | 0.50 | 0 | 1 | 0.34 | 0.48 | 0 | 1 | 0.20 | 0.41 | 0 | 1 |  | *** | * |
| % Neighbors with PCWR | 0.46 | 0.16 | 0.14 | 0.69 | 0.39 | 0.16 | 0.17 | 0.72 | 0.35 | 0.15 | 0.17 | 1 | *** | *** | ** |
| *External* |  |  |  |  |  |  |  |  |  |  |  |  |  |  | *** |
| Avg. annual max. temperature 1986-1990, °C | 22 | 2.7 | 10.4 | 26 |  |  |  |  | 22 | 3.6 | 14.7 | 30 | *** | *** |  |
| Avg. annual max. temperature 1992-1996, °C |  |  |  |  | 21 | 3.3 | 13.1 | 30 |  |  |  |  |  |  | *** |
| N |  | 116 |  |  |  | 100 |  |  |  | 69 |  |  |  |  |  |

Note: *** Significant at the 1 percent level, ** Significant at the 5 percent level, * Significant at the 10 percent level.

Table S5. Summary Statistics: 1991-2015 Panel, Waves of Adoption.

|  | **All California CWS** | | | | *Sig. Difference* | | |
| --- | --- | --- | --- | --- | --- | --- | --- |
| Variable | Mean | SD | Min | Max | *Repeated Cross-Section* | *Panel, 1991-2015* | *Panel, 2006-2015* |
| *Capacity* |  |  |  |  |  |  |  |
| Large utility (>10,000 people served) | 0.15 | 0.36 | 0 | 1 | *** | *** | *** |
| Service population | 14,179 | 96,746 | 12 | 3,935,257 | *** | *** | *** |
| % Unmetered service connections | 0.40 | 0.47 | 0 | 1 | *** | *** | *** |
| *Motivation* |  |  |  |  |  |  |  |
| Governance: Special district | 0.24 | 0.43 | 0 | 1 | *** | *** | *** |
| Governance: Private owner | 0.62 | 0.49 | 0 | 1 | *** | *** | *** |
| Governance: General-purpose government | 0.10 | 0.30 | 0 | 1 | *** | *** | *** |
| South-of-Delta water importer | 0.15 | 0.36 | 0 | 1 | *** | *** | *** |
| *External* |  |  |  |  |  |  |  |
| Avg. annual max. temperature 1986-1990, °C | 21.2 | 3.3 | 10 | 30.3 | *** | *** | *** |
| Avg. annual max. temperature 1992-1996, °C | 20.9 | 3.4 | 9.5 | 30.3 | *** | *** | *** |
| N | 2,818 |  |  |  |  |  |  |

Note: *** Significant at the 1 percent level, ** Significant at the 5 percent level, * Significant at the 10 percent level.

Table S6. Summary Statistics: All CWS in California.

|  | **California CWS,**  **serving 3,000 or more** | | | | *Sig. Difference* | | |
| --- | --- | --- | --- | --- | --- | --- | --- |
| Variable | Mean | SD | Min | Max | *Repeated Cross-Section* | *Panel, 1991-2015* | *Panel, 2006-2015* |
| *Capacity* |  |  |  |  |  |  |  |
| Large utility (>10,000 people served) | 0.63 | 0.48 | 0 | 1 | *** | *** | *** |
| Service population | 57,882 | 191,165 | 3,000 | 3,935,257 | *** | *** |  |
| % Unmetered service connections | 0.10 | 0.27 | 0 | 1 | *** | *** | *** |
| *Motivation* |  |  |  |  |  |  |  |
| Governance: Special district | 0.36 | 0.48 | 0 | 1 | ** |  |  |
| Governance: Private owner | 0.20 | 0.40 | 0 | 1 | *** | *** |  |
| Governance: General-purpose government | 0.38 | 0.48 | 0 | 1 | *** | *** | ** |
| South-of-Delta water importer | 0.31 | 0.46 | 0 | 1 | ** |  | *** |
| *External* |  |  |  |  |  |  |  |
| Avg. annual max. temperature 1986-1990, °C | 21.9 | 2.9 | 10.4 | 30.3 |  |  | *** |
| Avg. annual max. temperature 1992-1996, °C | 21.7 | 2.9 | 9.5 | 30.3 |  |  | *** |
| N | 676 |  |  |  |  |  |  |

Note: A service population of 3,000 or more customers is the threshold to be subject to metering requirements for new construction, as specified in Senate Bill SB 229, implemented in 1992.

*** Significant at the 1 percent level, ** Significant at the 5 percent level, * Significant at the 10 percent level.

Table S7. Summary Statistics: CWS in California serving 3,000 or more people.

|  | **First-Wave Adoption** | | | **Second-Wave Adoption** | | | | | | **Never Adopted** | | |
| --- | --- | --- | --- | --- | --- | --- | --- | --- | --- | --- | --- | --- |
|  | **Adopted PCWR during 1991-95** | | | **Adopted PCWR during 1997-2015** | | | | | | **Never adopted PCWR during 1991-2015** | | |
|  |  | (A1) |  |  | (A2) |  |  | (A3) |  |  | (A4) |  |
|  | All obs. | | | Exclude First Wave | | | All obs. | | | All obs. | | |
|  | ME |  | Std Err. | ME |  | Std Err. | ME |  | Std Err. | ME |  | Std Err. |
| *Capacity* |  |  |  |  |  |  |  |  |  |  |  |  |
| Large utility | -0.086 |  | 0.071 | 0.127 |  | 0.093 | 0.129 | ** | 0.064 | -0.046 |  | 0.062 |
| % Unmetered service connections^1^ | -0.901 | ** | 0.408 | -0.211 |  | 0.157 | -0.023 |  | 0.153 | 0.298 | *** | 0.110 |
| ln(Median household income) | 0.089 |  | 0.100 | 0.204 |  | 0.129 | 0.065 |  | 0.100 | -0.159 | * | 0.088 |
| *Motivation* |  |  |  |  |  |  |  |  |  |  |  |  |
| Governance: Special district | -0.009 |  | 0.058 | 0.008 |  | 0.076 | 0.015 |  | 0.059 | -0.012 |  | 0.050 |
| Governance: Private owner | -0.234 | ** | 0.091 | 0.368 | *** | 0.079 | 0.435 | *** | 0.103 | -0.185 | *** | 0.068 |
| % Neighbors with PCWR^2^ | 0.624 | *** | 0.188 | -0.008 |  | 0.281 | -0.389 | * | 0.202 | -0.264 |  | 0.170 |
| *External* |  |  |  |  |  |  |  |  |  |  |  |  |
| Avg. annual max. temperature 1986-1990, °C | 0.010 |  | 0.009 |  |  |  |  |  |  | 0.004 |  | 0.007 |
| Avg. annual max. temperature 1992-1996, °C |  |  |  | -0.011 |  | 0.010 | -0.012 |  | 0.009 |  |  |  |
| Log likelihood | -174 | | | -104 | | | -176 | | | -141 | | |
| LR χ^2^ | 37 | | | 21 | | | 17 | | | 33 | | |
| Prob> χ^2^ | 0.000 | | | 0.004 | | | 0.015 | | | 0.000 | | |
| McFadden's R^2^ | 0.095 | | | 0.092 | | | 0.047 | | | 0.105 | | |
| N | 285 | | | 169 | | | 285 | | | 285 | | |

Notes:

^1^ In the 1991-2015 panel, median percent of unmetered connections is calculated using all years available from SWRCB, 2013-2015.

^2^ The values of *% Neighbors with PCWR* represent the portion of other utilities with PCWRs in the First-Wave.

Delta method standard errors are reported for marginal effects.

* Statistically significant at the 10% level. ** Statistically significant at the 5% level. *** Statistically significant at the 1% level.

Model (A3) includes all observations (2nd wave adopters + 1st wave + Never adopt), while Model (A2) includes only 2nd wave + Never adopt.

**Table S8. Regression Results for 1991-2015 Panel, Marginal Effects.**

|  | **Adopted PCWR during 1991-95** | | | **Adopted PCWR during 1997-2015** | | | | | | **Never adopted PCWR during 1991-2015** | | |
| --- | --- | --- | --- | --- | --- | --- | --- | --- | --- | --- | --- | --- |
|  |  | (A5) |  |  | (A6) |  |  | (A7) |  |  | (A8) |  |
|  | All obs. | | | Exclude First Wave | | | All obs. | | | All obs. | | |
|  | ME |  | Std Err. | ME |  | Std Err. | ME |  | Std Err. | ME |  | Std Err. |
| *Capacity* |  |  |  |  |  |  |  |  |  |  |  |  |
| Large utility | -0.057 |  | 0.072 | 0.126 |  | 0.092 | 0.114 | * | 0.065 | -0.056 |  | 0.062 |
| % Unmetered service connections^1^ | -0.968 | ** | 0.430 | -0.211 |  | 0.156 | 0.000 |  | 0.153 | 0.313 | *** | 0.112 |
| ln(Median household income) | 0.231 | ** | 0.090 | 0.202 | * | 0.113 | -0.019 |  | 0.091 | -0.222 | *** | 0.078 |
| *Motivation* |  |  |  |  |  |  |  |  |  |  |  |  |
| Governance: Special district | -0.022 |  | 0.059 | 0.008 |  | 0.076 | 0.025 |  | 0.059 | -0.006 |  | 0.051 |
| Governance: Private owner | -0.209 | ** | 0.099 | 0.368 | *** | 0.079 | 0.419 | *** | 0.109 | -0.189 | *** | 0.065 |
| *External* |  |  |  |  |  |  |  |  |  |  |  |  |
| Avg. annual max. temperature 1986-1990, °C | 0.010 |  | 0.009 |  |  |  |  |  |  | 0.004 |  | 0.007 |
| Avg. annual max. temperature 1992-1996, °C |  |  |  | -0.011 |  | 0.010 | -0.013 |  | 0.009 |  |  |  |
| Log likelihood | -179 | | | -104 | | | -178 | | | -142 | | |
| LR χ^2^ | 27 | | | 21 | | | 14 | | | 31 | | |
| Prob> χ^2^ | 0.000 | | | 0.002 | | | 0.034 | | | 0.000 | | |
| McFadden's R^2^ | 0.069 | | | 0.092 | | | 0.037 | | | 0.098 | | |
| N | 285 | | | 169 | | | 285 | | | 285 | | |

Notes:

^1^ In the 1991-2015 panel, median percent of unmetered connections is calculated using all years available from SWRCB, 2013-2015.

Delta method standard errors are reported for marginal effects.

* Statistically significant at the 10% level. ** Statistically significant at the 5% level. *** Statistically significant at the 1% level.

Model (A7) includes all observations (2nd wave adopters + 1st wave + Never adopt), while Model (A6) includes only 2nd wave + Never adopt.

**Table S9.** Additional Regression Results for 1991-2015 Panel, Marginal Effects. (Excludes % Neighbors with PCWR)

|  | **First-Wave Adoption** | | | **Second-Wave Adoption** | | | | | | **Never Adopted** | | |
| --- | --- | --- | --- | --- | --- | --- | --- | --- | --- | --- | --- | --- |
|  | **Adopted PCWR during 1991-95** | | | **Adopted PCWR during 1997-2015** | | | | | | **Never adopted PCWR during 1991-2015** | | |
|  |  | (A9) |  |  | (A10) |  |  | (A11) |  |  | (A12) |  |
|  | All obs. | | | Exclude First Wave | | | All obs. | | | All obs. | | |
|  | ME |  | Std Err. | ME |  | Std Err. | ME |  | Std Err. | ME |  | Std Err. |
| *Capacity* |  |  |  |  |  |  |  |  |  |  |  |  |
| Large utility | -0.044 |  | 0.072 | 0.147 |  | 0.093 | 0.120 | * | 0.064 | -0.069 |  | 0.064 |
| % Unmetered service connections^1^ | -1.014 | ** | 0.437 | -0.256 |  | 0.156 | -0.006 |  | 0.150 | 0.357 | *** | 0.111 |
|  |  |  |  |  |  |  |  |  |  |  |  |  |
| *Motivation* |  |  |  |  |  |  |  |  |  |  |  |  |
| Governance: Special district | -0.014 |  | 0.059 | 0.018 |  | 0.077 | 0.027 |  | 0.059 | -0.014 |  | 0.051 |
| Governance: Private owner | -0.221 | ** | 0.097 | 0.365 | *** | 0.081 | 0.423 | *** | 0.108 | -0.185 | *** | 0.067 |
| South-of-Delta water importer | 0.112 | * | 0.062 | 0.078 |  | 0.088 | -0.036 |  | 0.060 | -0.094 | * | 0.053 |
| *External* |  |  |  |  |  |  |  |  |  |  |  |  |
| Avg. annual max. temperature 1986-1990, °C | 0.007 |  | 0.009 |  |  |  |  |  |  | 0.006 |  | 0.008 |
| Avg. annual max. temperature 1992-1996, °C |  |  |  | -0.013 |  | 0.010 | -0.012 |  | 0.009 |  |  |  |
| Log likelihood | -181 | | | -105 | | | -178 | | | -145 | | |
| LR χ^2^ | 24 | | | 19 | | | 14 | | | 26 | | |
| Prob> χ^2^ | 0.001 | | | 0.004 | | | 0.030 | | | 0.000 | | |
| McFadden's R^2^ | 0.062 | | | 0.083 | | | 0.038 | | | 0.082 | | |
| N | 285 | | | 169 | | | 285 | | | 285 | | |

Notes: Delta method standard errors are reported for marginal effects.

* Statistically significant at the 10% level. ** Statistically significant at the 5% level. *** Statistically significant at the 1% level.

Model (A11) includes all observations (2nd wave adopters + 1st wave + Never adopt), while Model (A10) includes only 2nd wave + Never adopt.

Table S10. Additional Regression Results for 1991-2015 Panel, Marginal Effects. (Includes South-of-Delta water importer)

1. Year 1996 is not observed for most utilities in the dataset since it was not a survey year for AWWA and B&V. [↑](#footnote-ref-1)
2. Since this break point is at the beginning of the study period, a Supremum Wald test cannot be used to test for a structural break, using symmetric trimming of 15%. [↑](#footnote-ref-2)
